# ﻿ *Ligusticopsis shaniana*, a new combination separated from *Peucedanum* sensu lato (Apiaceae) on the basis of morphological and phylogenetic evidence

**DOI:** 10.3897/phytokeys.263.163085

**Published:** 2025-09-29

**Authors:** Chang-Kun Liu, Bo-Ni Song, Jia-Qing Lei, Xing-Jin He, Song-Dong Zhou

**Affiliations:** 1 Key Laboratory of Bio-Resources and Eco-Environment of Ministry of Education, College of Life Sciences, Sichuan University, Chengdu 610065, China Sichuan University Chengdu China; 2 College of Resources Environment and Chemistry, Chuxiong Normal University, Chuxiong 675000, China Chuxiong Normal University Chuxiong China

**Keywords:** China, morphology, *Peucedanum* sensu lato, phylogeny

## Abstract

The genus *Peucedanum* sensu lato is one of the most taxonomically difficult taxa within Apiaceae, including many medically and economically valuable plants. Although previous studies performed several taxonomic revisions for this genus, the taxonomic position of many taxa in the genus still has not been satisfactorily resolved, especially those endemic to China. In this study, to clarify the taxonomic position of *Peucedanum shanianum*, we performed comprehensive morphological and molecular analyses for this species. Morphological comparisons showed that *Peucedanum shanianum* has pinnate leaves, both pinnate and linear bracteoles, and numerous vittae in the mericarp commissure and each furrow, which are extremely similar to the genus *Ligusticopsis* but clearly different from *Peucedanum* sensu stricto, which has ternate leaves, linear-subulate or filiform bracteoles, a single vitta in a furrow, and two vittae on the commissure in a mericarp. However, mericarps with filiform dorsal ribs and linear bracts can easily distinguish *Peucedanum shanianum* from other *Ligusticopsis* members. Furthermore, phylogenetic analyses based on plastome data and ITS sequences indicated that *Peucedanum shanianum* is only distantly related to *Peucedanum* sensu stricto members but nested in *Ligusticopsis*. Therefore, combining the evidence of morphology and phylogeny, we suggest that *Peucedanum shanianum* should be transferred to *Ligusticopsis*, and a new combination (*Ligusticopsis shaniana*) is proposed.

## ﻿Introduction

*Peucedanum* sensu lato, the backbone member of the subfamily Apioideae of the family Apiaceae with 100–120 species, is distributed in Eurasia and South Africa (and partly Australia). The genus is characterized by dorsally compressed mericarps with slightly elevated dorsal ribs, narrowly winged lateral ribs, and a broad commissure (Sheh 1992; Spalik et al. 2004; Sheh and Watson 2005). It is one of the most taxonomically difficult genera, largely due to the varied morphological features of life form, leaf structure, and chemical constituents (Shneyer et al. 2003). Previous molecular phylogenetic studies based on a few molecular markers failed to recognize *Peucedanum* sensu lato as a monophyletic group, of which only a few European species–with one species (*P. morisonii* Besser & Schult.) extending to East Asia–were consistently clustered with *P. officinale* L., the type species of this genus (Downie et al. 2000; Spalik et al. 2004; Valiejo-Roman et al. 2006; Feng et al. 2009; Zhou et al. 2009, 2020; Liu et al. 2022). Hence, *Peucedanum* sensu stricto was adopted to include only seven species (Pimenov 2018), characterized by ternate leaves, linear leaflets, yellow petals, dorsally compressed and glabrous mericarps with 1 vitta in each furrow and 2 on the commissure. The remaining members of *Peucedanum* sensu lato were separated into restored or newly established genera or transferred into other genera. For example, Reduron et al. (1997) restored the genera *Cervaria* Wolf, *Imperatoria* L., *Oreoselinum* Mill., *Pteroselinum* Rchb., *Thysselinum* Adans., *Xanthoselinum* Schur, and *Holandrea* Reduron, Charpin & Pimenov from *Peucedanum* sensu lato based on morphological and phytochemical evidence. Winter et al. (2008) established three new genera (*Afrosciadium* P.J.D. Winter, *Nanobubon* Magee, and *Notobubon* B.-E. van Wyk) to accommodate the African taxa and transferred 24 species of *Peucedanum* sensu lato into *Afroligusticum* C. Norman, *Cynorhiza* Eckl. & Zeyh., and *Lefebvrea* A. Rich. based on morphological and molecular data. Song et al. (2025) recovered two monophyletic clades within *Peucedanum* sensu lato based on genomic skimming data and integrated evidence from morphology, plastome, and chromosome numbers to establish two new genera (*Shanopeucedanum* B.N. Song, C.K. Liu & X.J. He and *Sinopeucedanum* B.N. Song, C.K. Liu & X.J. He). Although several studies focused on the taxonomic revision of *Peucedanum* sensu lato have been performed, the systematic positions of numerous species of this genus still have not been satisfactorily resolved, especially for those endemic to China. Therefore, it is necessary to conduct the revision of these taxa to improve the taxonomic system of subfamily Apioideae.

**Figure 1.**
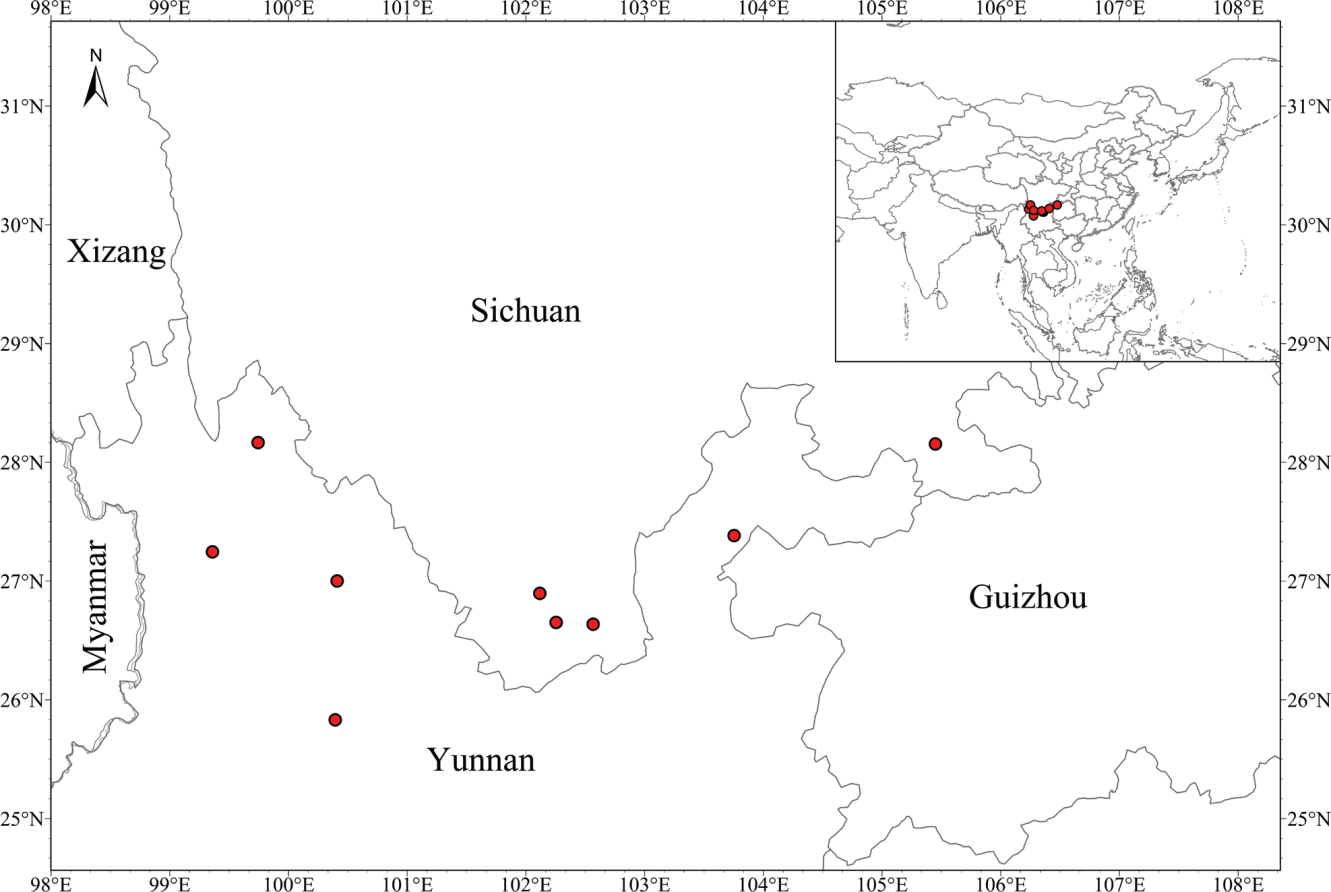
Distribution of *Peucedanum shanianum*.

This study focused on *Peucedanum shanianum* F.L. Chen & Y.F. Deng, which is endemic to China (Fig. 1), growing in scrub, grassy slopes, or rock crevices at elevations of 2,000–3,000 m (Sheh and Watson 2005). *Peucedanum shanianum* was recognized as a member of the genus *Peucedanum* sensu lato because of its dorsally compressed mericarps with slightly elevated dorsal ribs and narrowly winged lateral ribs (Shan et al. 1986). However, Shan et al. (1986) noted that this species is significantly similar to *Ligusticopsis brachyloba* (Franch.) Leute in overall morphology. Thus, the taxonomic position of *Peucedanum shanianum* is unclear and requires re-evaluation.

## ﻿Materials and methods

### ﻿Taxon sample and morphological observations

Three populations of *Peucedanum shanianum* were collected from Yunnan, China (Table 1), and fresh leaves were dried with silica gel. Features of habitat and morphology for this species were observed in the field. Its mericarp was examined using a stereomicroscope (Nikon SMZ25, Japan) at Sichuan University. Images of the type specimens were obtained from the Chinese Virtual Herbarium (https://www.cvh.ac.cn). In addition, morphological characters of other *Ligusticopsis* Leute members, including *L. hispida* (Franch.) Lavrova & Kljuykov, *L. involucrata* (Franch.) Lavrova ex Pimenov, *L. rechingerana* Leute, *L. modesta* (Diels) Leute, *L. integrifolia* (H. Wolff) Leute, *L. capillacea* Leute, *L. scapiformis* (H. Wolff) Leute, *L. wallichiana* (DC.) Pimenov & Kljuykov, and *L. brachyloba* (Franch.) Leute, were obtained and summarized from the study of Li et al. (2022) by our team.

**Table 1.** ITS sequences newly obtained in this study with voucher information and GenBank accession.

Taxa	Specimen voucher	Locality	GenBank accession
*P. shanianum*	L20210702	Tongle, Weixi, Yunnan, China	(ITS) ON124710
*P. shanianum*	L20210703	Jiantang, Shangri-la, Yunnan, China	(ITS) ON124711
*P. shanianum*	L20210704	Xiaozhongdian, Shangri-la, Yunnan, China	(ITS) ON124712

### ﻿DNA extraction, ITS amplification, and sequencing

Total genomic DNA was isolated from silica-dried leaves with a plant genomic DNA kit (Cwbio Biosciences, Beijing, China). The universal primers ITS4 (5′-TCC TCC GCT TAT TGA TAT GC-3′) and ITS5 (5′-GGA AGT AAA AGT CGT AAC AAG G-3′) were used to amplify the nuclear ribosomal internal transcribed spacer (ITS) region (White et al. 1990). Amplification of ITS was conducted by initial denaturation for 3 min at 94 °C, followed by 30 cycles of 45 s at 94 °C, 70 s at 54 °C, and 90 s at 72 °C, with a final extension of 10 min at 72 °C. The PCR products were then sequenced by Sangon Biotech (Shanghai, China).

### ﻿Plastome sequencing and assembly

We used 1% agarose gel electrophoresis to test the quality and quantity of DNA, and then the high-quality DNA was sequenced on the Illumina HiSeq2500 platform at Novogene (Beijing, China) according to standard Illumina sequencing protocols, applying paired-end 150-bp reads with an average insert size of 300–400 bp. Quality control of the raw data was performed by the software fastP v0.15.0 (-n 10 and -q 15) (Chen et al. 2018), which yielded at least 5 GB of clean reads for *Peucedanum shanianum*. NOVOPlasty v2.7.2 (Dierckxsens et al. 2017) was employed to assemble a complete plastome with the default parameters, using the rbcL sequence extracted from the plastome of *Ligusticopsis brachyloba* (Franch.) Leute (MN204661) as the seed. The assembled plastome was annotated initially with the program DOGMA (Wyman et al. 2004) and then examined using Geneious R11 (Biomatters, Ltd., Auckland, New Zealand). The circular plastome map was displayed using the online program CHLOROPLOT (https://irscope.shinyapps.io/Chloroplot/) (Zheng et al. 2020).

### ﻿Phylogenetic analyses

To confirm the taxonomic position of *Peucedanum shanianum*, phylogenetic analyses were performed based on two datasets: data 1 consisted of 41 plastome sequences, and data 2 consisted of 41 ITS sequences of Apiaceae (Suppl. material 1: table S1). *Chamaesium mallaeanum* Farille & S.B. Malla and *Chamaesium viridiflorum* (Franch.) H. Wolff ex R.H. Shan were selected as outgroups according to previous studies (Clarkson et al. 2021; Wen et al. 2021). Forty-one plastome sequences and 41 ITS sequences were aligned using the software MAFFT v7.221 (Katoh and Standley 2013) and adjusted manually when necessary. Two aligned matrices were subjected to Maximum Likelihood (ML) analyses and Bayesian Inference (BI). The ML analysis was conducted using the program RAxML v8.2.8 (Stamatakis 2014) with 1,000 replicates and the GTRGAMMA model as suggested in the RAxML manual. The BI analysis was performed using MrBayes v3.2.7 (Ronquist et al. 2012) with the best-fit substitution model (GTR+I+G) determined by Modeltest v3.7 (Posada and Crandall 1998) for both datasets. Two independent Markov chains were run for 1,000,000 generations, sampling every 100 generations. The initial 25% of trees were discarded as burn-in, and the remaining trees were used to yield the consensus tree. Finally, the software FigTree v1.4.2 (Rambaut and Drummond 2015) and the online tool iTOL (https://itol.embl.de/itol.cgi) (Letunic and Bork 2007) were used to edit and visualize the phylogenetic trees.

## ﻿Results

### ﻿Morphological observations

**Figure 2.**
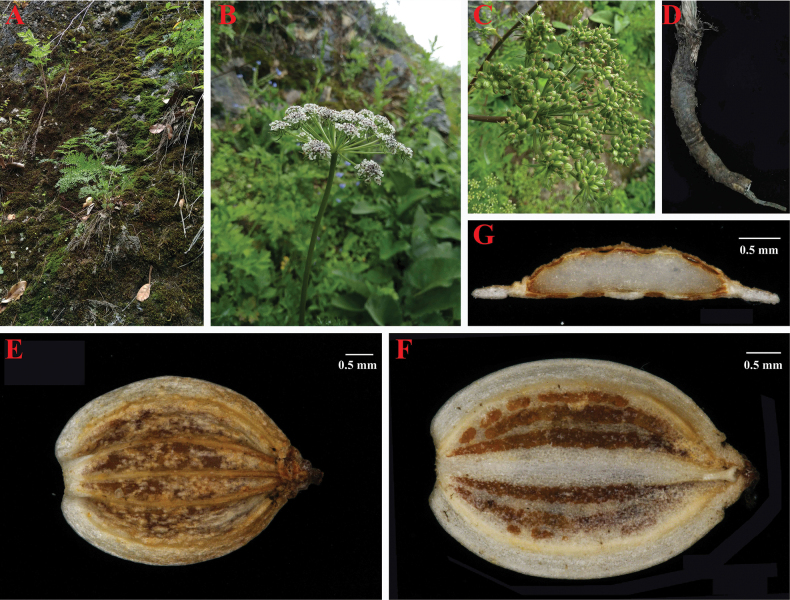
Illustrations of *Peucedanum shanianum*. A. Habit; B. Umbel with bracts and bracteoles; C. Young mericarp with calyx teeth; D. Root with fibrous remnant sheaths; E. Dorsal of mericarp; F. Commissure of mericarp; G. Cross-section of mericarp.

In the wild, we observed that *Peucedanum shanianum* is a perennial herb that grows in scrub, grassy slopes, or rock crevices at elevations of 2,000–3,360 m (Fig. 2A). It has pinnate leaves, linear leaflets, both pinnate and linear bracteoles, conspicuous calyx teeth, and a stem base clothed in fibrous remnant sheaths (Fig. 2B–D). In addition, we found that *Peucedanum shanianum* has dorsally compressed mericarps with filiform dorsal ribs, narrowly winged lateral ribs, and numerous vittae in the commissure and each furrow (Fig. 2E–G). Furthermore, we compared and summarized the morphological differences between *Peucedanum shanianum* and other *Ligusticopsis* members (Table 2).

**Table 2.** Comparison of morphological characters between *Peucedanum shanianum* and verified *Ligusticopsis* members.

Characteristics	*P. shanianum*	*L. brachyloba*	*L. capillacea*	*L. hispida*	*L. integrifolia*	*L. involucrata*	*L. modesta*	*L. rechingerana*	*L. scapiformis*	*L. wallichiana*
Stem (base)	Fibrous remnant sheaths	Fibrous remnant sheaths	Fibrous remnant sheaths	Fibrous remnant sheaths	Fibrous remnant sheaths	Fibrous remnant sheaths	Fibrous remnant sheaths	Fibrous remnant sheaths	Fibrous remnant sheaths	Fibrous remnant sheaths
Leaf	Pinnate	Pinnate	Pinnate	Pinnate	Pinnate	Pinnate	Pinnate	Pinnate	Pinnate	Pinnate
Ultimate segments of leaf	Linear	Linear	Linear-lanceolate	Lanceolate	Oblong-ovate or lanceolate	Oblong-ovate	Linear or lanceolate	Ovate	Linear to lanceolate	Linear
Bracts	Linear	Pinnate	Pinnate	Pinnate	Pinnate and linear together	Pinnate	Pinnate	Pinnate	Pinnate	Pinnate
Bracteoles	Pinnate and linear together	Pinnate and linear together	Pinnate	Pinnate	Pinnate	Pinnate	Pinnate	Pinnate	Pinnate	Pinnate and linear together
Mericarp	Elliptic	Elliptic	Ovate	Elliptic	Elliptic to ovate	Elliptic	Elliptic to oblong	Elliptic to ovate	Elliptic to ovate	Elliptic to orbicular
Calyx teeth	Conspicuous	Conspicuous	Conspicuous	Conspicuous	Conspicuous	Conspicuous	Conspicuous	Conspicuous	Conspicuous	Conspicuous
Dorsal compression	Strong	Strong	Strong	Strong	Strong	Strong	Strong	Strong	Strong	Strong
Dorsal ribs	Filiform	Keeled	Filiform	Filiform	Filiform	Filiform	Filiform	Filiform	Filiform	Keeled
Lateral ribs	Winged	Winged	Winged	Winged	Winged	Winged	Winged	Winged	Winged	Winged
Vittae per furrow	1~2	2~3	1~3	1~3	1~3	1~3	3~4	1~3	1~4	1~3
Commissural vittae	6	4~6	6	6	6	6	8	6	4~6	4~8

### ﻿Plastome feature of *Peucedanum shanianum*

**Figure 3.**
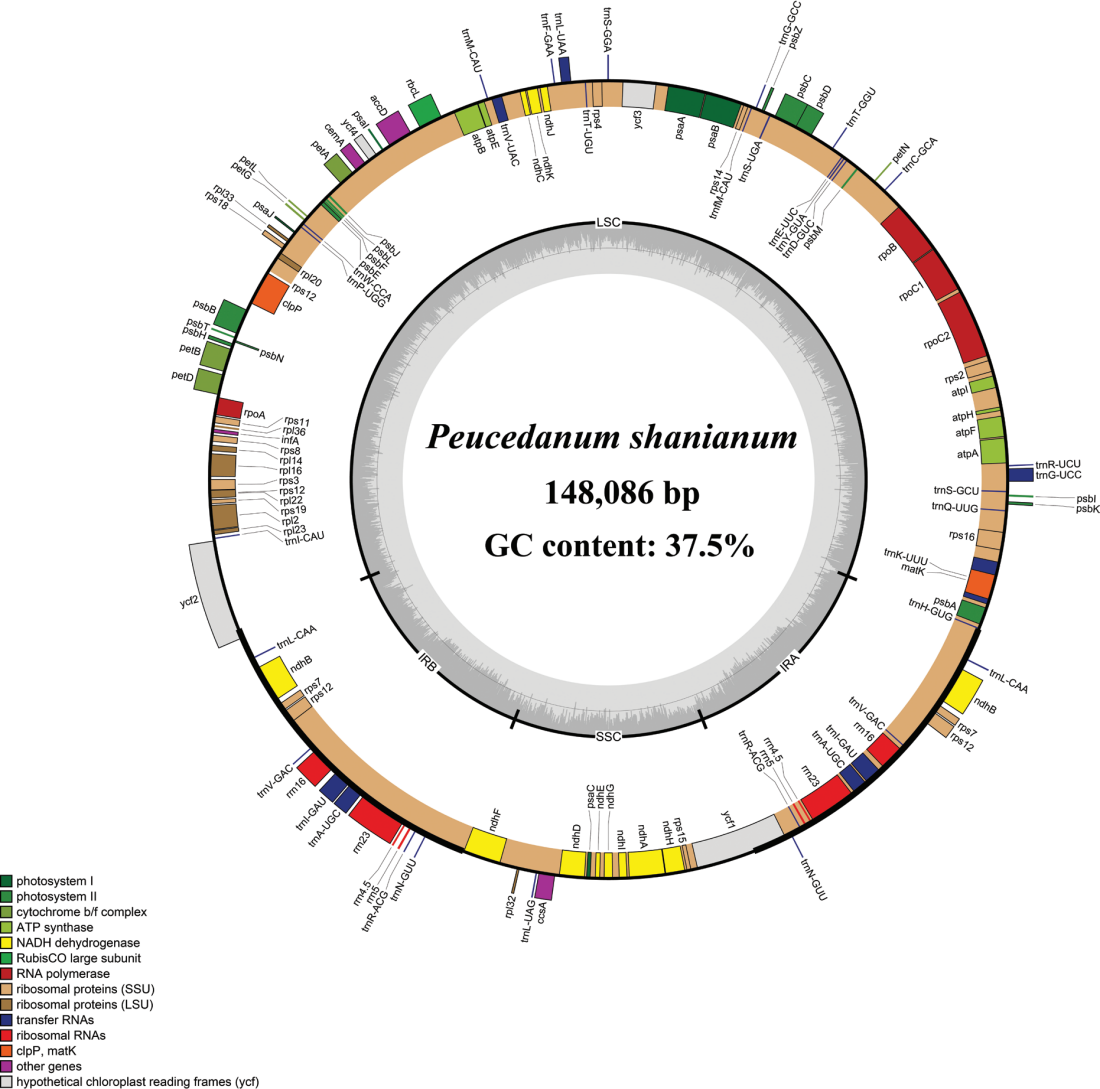
Plastome map of *Peucedanum shanianum*. The genes exhibited outside of the circle are transcribed clockwise, while those inside are counterclockwise. The genes belonging to different functional groups are color-coded.

The plastome of *Peucedanum shanianum* exhibited a typical quadripartite structure, containing one large single-copy region (LSC), one small single-copy region (SSC), and two inverted repeat regions (IRs). The plastome size was 148,086 bp (LSC: 92,153 bp; SSC: 17,581 bp; IR: 19,176 bp) and encoded 126 genes, including 82 protein-coding genes, 36 tRNAs, and 8 rRNAs. The total GC content was 37.5%, and the IR regions had the highest GC content (44.1%) compared with the LSC region (36.0%) and SSC region (30.9%) (Fig. 3).

### ﻿Phylogenetic inferences

**Figure 4.**
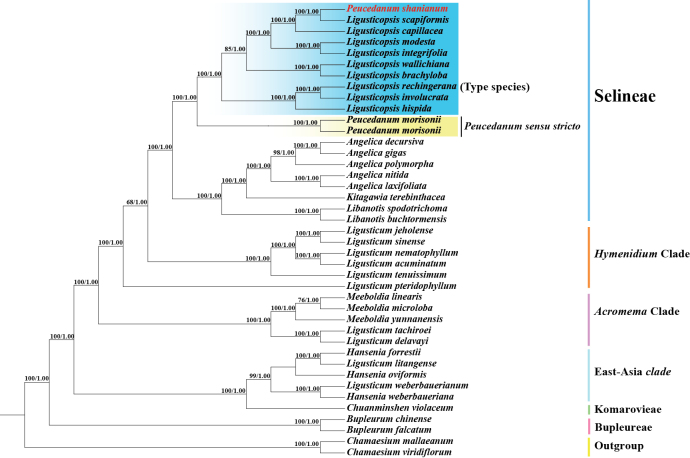
Phylogeny of *Peucedanum shanianum* inferred from maximum likelihood (ML) and Bayesian inference (BI) analyses based on plastome data. Numbers represent maximum likelihood bootstrap values (BS) and Bayesian posterior probabilities (PP).

**Figure 5.**
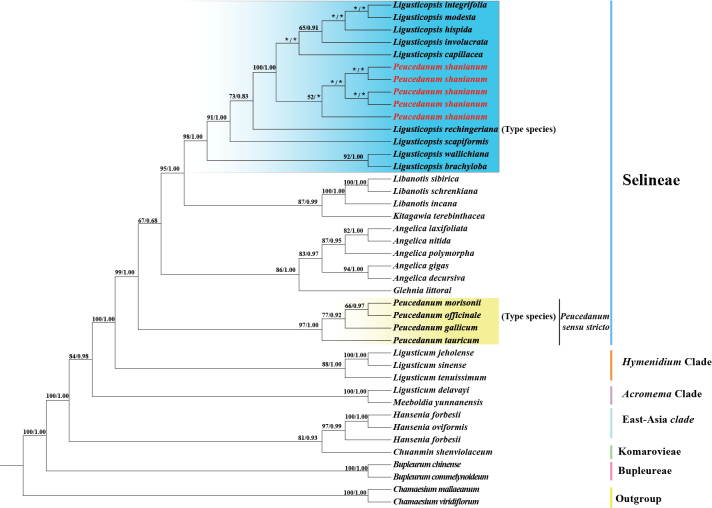
Phylogeny of *Peucedanum shanianum* inferred from maximum likelihood (ML) and Bayesian inference (BI) analyses based on ITS sequences. Numbers represent maximum likelihood bootstrap values (BS) and Bayesian posterior probabilities (PP). * means that the value of the node is less than 50%.

The plastome-based and ITS-based phylogenetic trees of ML and BI analyses yielded identical topologies, respectively (Figs 4, 5). Although the tree topologies of the plastome data and ITS sequences were incongruent, both strongly showed that *Peucedanum shanianum* was not clustered with *Peucedanum* sensu stricto but nested in *Ligusticopsis* (plastome: BS = 100, PP = 1.00; ITS: BS = 98, PP = 1.00) (Figs 4, 5). In the plastome-based phylogenetic trees, *Peucedanum shanianum* was sister to *Ligusticopsis scapiformis* with high support (BS = 100, PP = 1.00) (Fig. 4). In contrast, in the ITS-based phylogenetic trees, five accessions of *Peucedanum shanianum* were recovered as a monophyletic group, but the relationship between *Peucedanum shanianum* and other *Ligusticopsis* species was not resolved in this dataset (Fig. 5).

## ﻿Discussion

*Peucedanum shanianum* was identified as a member of the genus *Peucedanum* because of the dorsally compressed mericarps with filiform dorsal ribs and narrowly winged lateral ribs, which were also detected in *Peucedanum* sensu stricto members (Pimenov 2018). However, other morphological characteristics of *Peucedanum shanianum*, such as pinnate leaves, both pinnate and linear bracteoles, and numerous vittae in the mericarp commissure and each furrow, were significantly different from *Peucedanum* sensu stricto species, which have ternate leaves, linear-subulate or filiform bracteoles, a single vitta in each furrow, and two vittae on the commissure in the mericarp (Pimenov 2018). Hence, accommodating *Peucedanum shanianum* in *Peucedanum* sensu stricto is inappropriate based on the above-mentioned features.

In addition, our phylogenetic analyses based on plastome data and ITS sequences robustly supported that *Peucedanum shanianum* falls into the genus *Ligusticopsis* with high support. The affinity between *Peucedanum shanianum* and *Ligusticopsis* was also supported by shared morphological features, such as the stem base clothed in fibrous remnant sheaths, pinnate leaves, linear leaflets, pinnate and linear bracteoles, conspicuous calyx teeth, dorsally compressed mericarps with filiform dorsal ribs and narrowly winged lateral ribs, and numerous vittae in the commissure and each furrow. However, the mericarps with filiform dorsal ribs and linear bracts can easily distinguish *Peucedanum shanianum* from other *Ligusticopsis* members (Table 2). Therefore, phylogenetic analyses and morphological features robustly support that *Peucedanum shanianum* should be transferred to *Ligusticopsis* as an independent species, and a new combination, *Ligusticopsis shaniana*, is required.

### ﻿Taxonomic treatment

#### ﻿ Ligusticopsis shaniana

Taxon classificationPlantaeApialesApiaceae

(F.L. Chen & Y.F. Deng) C.K. Liu & X.J. He comb. nov.

78ED9C00-B868-5043-B97A-F292C24BF63B

urn:lsid:ipni.org:names:77369839-1

Fig. 6

≡ Peucedanum rubricaule R.H. Shan & M.L. Sheh (1986, p. 305), nom. illeg., non (Boiss.) Baill. (1879)
≡ Peucedanum shanianum F.L. Chen & Y.F. Deng (2019, p. 267)

##### Diagnosis.

This species can be easily distinguished from other *Ligusticopsis* members by the mericarps with filiform dorsal ribs and linear bracts.

**Figure 6.**
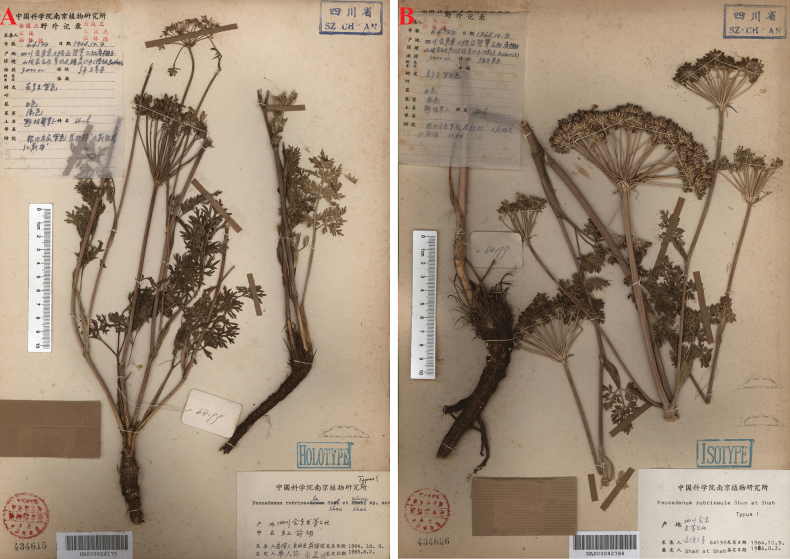
Specimen images of *Peucedanum shanianum*. A. Holotype; B. Isotype.

##### Description.

Perennial herbs, 30–80 cm. Stem solitary or several, erect, purplish, hollow, pubescent, base clothed with fibrous remnant sheaths. Basal petioles with purplish, puberulous, and scarious-margined sheaths; leaf blade triangular-ovate, 3-pinnatisect; pinnae 3–5 pairs, pinnules 3–4 pairs; ultimate segments linear, 3–10 × 1–1.6 mm, both surfaces glabrous, apex mucronate; cauline leaves reduced upwards, petioles wholly sheathing. Umbels ca. 10 cm across; bracts 6–10, linear, 10–15 × ca. 0.5 mm, puberulous; rays numerous, 24–40, subequal, 3–5 cm; bracteoles ca. 10, both pinnate and linear; umbellules ca. 20-flowered. Calyx teeth conspicuous, triangular, acute. Mericarp elliptic, 4–6 × 3–4 mm, glabrous; dorsal ribs filiform, lateral ribs narrowly winged, wings rather thick; vittae 1–2 in each furrow, 4–6 on commissure.

##### Distribution and habitat.

*Ligusticopsis shaniana* is distributed in southwestern China (southern Sichuan, northwestern Yunnan). It grows in scrub, grassy slopes, or rock crevices, at altitudes of 2,000–3,360 m a.s.l.

##### Specimens examined.

China, Sichuan: • Yanyuan County, Yousuo, 2750 m a.s.l., 11 Aug. 1963, Q. L. Zhang 1165 (NAS). China, Yunnan: • Shangri-la County, Napa Sea, 3300 m a.s.l., 14 July 2008, Q. E. Yang & Q. Yuan Yang QE1843 (KUN); • Shangri-la County, Xiaozhongdian, 3214 m a.s.l., 15 Sept. 2008, Q. S. Yang, Y. W. Xin & T. Su ZhouZK-07ZX-0380 (KUN); • Shangri-la County, Jiantang, 3360 m a.s.l., 15 July 2021, C. K. Liu & J. Q. Lei L20210703 (SZ); • Shangri-la County, Xiaozhongdian, 3200 m a.s.l., 16 July 2021, C. K. Liu & J. Q. Lei L20210704 (SZ); • Weixi County, Tongle, 3306 m a.s.l., 14 July 2021, C. K. Liu & J. Q. Lei L20210702 (SZ).

##### Type.

China, Sichuan: • Huidong, 2,000–3,000 m a.s.l., 9 Oct. 1964, J. S. Yue et al. 64199 (holotype: NAS).

## Supplementary Material

XML Treatment for Ligusticopsis shaniana

## ﻿Additional information

### Use of AI

No use of AI was reported.

### Author ORCIDs

Chang-Kun Liu https://orcid.org/0000-0001-6325-5463

Bo-Ni Song https://orcid.org/0000-0002-2274-3791

Jia-Qing Lei https://orcid.org/0000-0002-3976-1136

Xing-Jin He https://orcid.org/0000-0003-2064-0112

Song-Dong Zhou https://orcid.org/0000-0002-1161-8573

### Data availability

The three newly sequenced ITS fragments and plastome have been submitted to NCBI with accession numbers ON124710–ON124712 and PX398768 respectively. The voucher specimens of Peucedanum shanianum were deposited at the herbarium of Sichuan University (Chengdu, China).

## Supplementary materials

Supplementary material 1 Sequences of plastomes and ITS obtained from NCBI Data type xlsx https://binary.pensoft.net/file/1426482This dataset is made available under the Open Database License (http://opendatacommons.org/licenses/odbl/1.0/). The Open Database License (ODbL) is a license agreement intended to allow users to freely share, modify, and use this Dataset while maintaining this same freedom for others, provided that the original source and author(s) are credited.Chang-Kun Liu, Bo-Ni Song, Jia-Qing Lei, Xing-Jin He, Song-Dong Zhou
